# Challenges and Opportunities of Therapies Targeting Early Life Immunity for Pediatric HIV Cure

**DOI:** 10.3389/fimmu.2022.885272

**Published:** 2022-07-13

**Authors:** Stella J. Berendam, Ashley N. Nelson, Bhrugu Yagnik, Ria Goswami, Tiffany M. Styles, Margaret A. Neja, Caroline T. Phan, Sedem Dankwa, Alliyah U. Byrd, Carolina Garrido, Rama R. Amara, Ann Chahroudi, Sallie R. Permar, Genevieve G. Fouda

**Affiliations:** ^1^ Duke Human Vaccine Institute, Duke University School of Medicine, Durham, NC, United States; ^2^ Department of Pediatrics, Duke University School of Medicine, Durham, NC, United States; ^3^ Department of Microbiology and Immunology, Emory Vaccine Center, Yerkes National Primate Research Center, Emory University, Atlanta, GA, United States; ^4^ Department of Pediatrics, Weill Cornell Medicine, New York, NY, United States; ^5^ Department of Pediatrics, Emory University School of Medicine, Atlanta, GA, United States; ^6^ Center for Childhood Infections and Vaccines of Children’s Healthcare of Atlanta and Emory University, Atlanta, GA, United States

**Keywords:** pediatric HIV, cure, early life immunity, immune-based therapies, HIV cure strategies

## Abstract

Early initiation of antiretroviral therapy (ART) significantly improves clinical outcomes and reduces mortality of infants/children living with HIV. However, the ability of infected cells to establish latent viral reservoirs shortly after infection and to persist during long-term ART remains a major barrier to cure. In addition, while early ART treatment of infants living with HIV can limit the size of the virus reservoir, it can also blunt HIV-specific immune responses and does not mediate clearance of latently infected viral reservoirs. Thus, adjunctive immune-based therapies that are geared towards limiting the establishment of the virus reservoir and/or mediating the clearance of persistent reservoirs are of interest for their potential to achieve viral remission in the setting of pediatric HIV. Because of the differences between the early life and adult immune systems, these interventions may need to be tailored to the pediatric settings. Understanding the attributes and specificities of the early life immune milieu that are likely to impact the virus reservoir is important to guide the development of pediatric-specific immune-based interventions towards viral remission and cure. In this review, we compare the immune profiles of pediatric and adult HIV elite controllers, discuss the characteristics of cellular and anatomic HIV reservoirs in pediatric populations, and highlight the potential values of current cure strategies using immune-based therapies for long-term viral remission in the absence of ART in children living with HIV.

## Introduction

In 2020, approximately 150,000 children were newly infected with HIV and 1.7 million children (<15 years of age) were living with HIV worldwide (1). Most of these children (˜90%) live in sub-Saharan Africa and were infected perinatally or during the breastfeeding period. Although antiretroviral therapy (ART) has significantly reduced the rate of vertical transmission, infant infections continue to occur due to incident of maternal HIV infections during pregnancy or breastfeeding, poor maternal access to ART treatment, challenges in maternal ART adherence, and lack of viral suppression in mothers receiving ART (2). Early ART treatment reduces mortality and improves clinical outcomes in infants with HIV. Thus, the current treatment guidelines recommend initiation of ART to infected infants as early as possible, regardless of clinical or immunological outcomes (3). Studies have shown that early ART initiation within the first months of life in infants with HIV suppresses viral replication and can result in a significantly smaller latent viral reservoir, preservation of CD4+ T cell counts, and decreased immune dysregulation (4–7). However, ART is not curative and does not eliminate the establishment of persistent latent viral reservoirs, which contribute to viral rebound when treatment is interrupted (8).

Previous studies have also reported that early ART initiation in infants with HIV is associated with a lack of circulating HIV-specific antibodies and T cell responses, likely due to low levels of circulating viral antigen and suppression of viral replication (6, 9–11). In these studies, approximately 36-46% of infants with perinatal HIV infection who were treated with ART within the first year of life became seronegative (9, 11–13). In a more recent study, Cotugno et al. reported that while these early ART-treated perinatally infected infants are seronegative, *in vitro* stimulation of the memory B cell population derived from peripheral blood mononuclear cells (PBMCs) with HIV peptides induced HIV-specific antibody responses, suggesting persistence of HIV-specific humoral memory despite the seronegative status (13). Interestingly, *in vitro* stimulation induced higher expression of transcriptional signature profiles of genes related to antibody production (PRDM1) and T-B cells cognate stimulation (CXCR4, IL21R) in early ART-treated infants who were seropositive compared to their seronegative counterparts, suggesting a truncated process of HIV-specific B cell maturation in seronegative children (13). Taken together these results suggest that while early ART initiation is beneficial for controlling viral replication, rapid elimination of viral antigen results in qualitatively distinct HIV-specific memory responses. Additional studies are needed to further define the impact of the timing of ART initiation on persistence of HIV-specific cellular and humoral immune responses in children and to define whether the distinct profiles of HIV-specific memory T and B cells in children who become seronegative has an impact on the potential of immunotherapies to promote cure.

The overall goal of this review is to discuss current knowledge of early life immunity that can be harnessed to develop future strategies aimed to achieve HIV remission in pediatric settings. Herein, we compare the characteristics of innate and adaptive immune cells in pediatric versus adult elite controllers, discuss the characteristics of cellular and anatomic HIV reservoirs in pediatric populations, and highlight the potential of novel immune-based interventions that have been studied in adults with HIV or in preclinical models to eliminate persistent latently infected viral reservoirs in children living with HIV.

## Immune Profiles of Pediatric and Adult HIV Elite Controllers

HIV elite controllers (ECs) are a rare group of individuals with HIV that have the capacity to spontaneously control viral load without treatment (14). Specifically, these individuals maintain a viral load of <50 copies/ml at least three times over the course of a year (15) and do not demonstrate CD4+ T cell depletion (16). These individuals are distinguished from long-term non progressors (LTNPs) who have low-to-moderate viremia (<5000 RNA copies/ml) but preserved CD4+ T cell numbers (15–17). ECs make up ~0.5% of all adults with HIV and the number is at least six times lower in children with HIV (~0.08%) (18, 19). While most ECs eventually lose control of viremia, they provide a model for natural suppression of HIV replication that can guide immunotherapeutic interventions towards long-term ART-free virus control. Herein, we examine and compare the adaptive and innate immune cell profiles of pediatric and adult ECs to guide development of immune-based strategies for latent reservoir clearance and HIV remission tailored to the pediatric settings.

### Cellular Immunity

#### CD8+ T Cells

The CD8+ T cell response has been identified as a distinct and crucial feature in HIV ECs (20). Both pediatric and adult ECs, compared to their progressor counterparts, possess a higher frequency of HIV Gag-specific, polyfunctional CD8+ T cells capable of cytokine release (IFN-g, TNF-a and IL-2), degranulation, and release of perforin and granzyme B (19, 21). Notably, in pediatric elite controllers (PECs), this polyfunctional population of HIV-specific CD8+ T cells is higher than in non-progressors, even though the overall production of cytokines is similar between these groups (19). Additionally, in line with previous observations in adults, pediatric progressors (PPs) had higher magnitude Nef-specific CD8+ T-cell responses compared to ECs (19). Additionally, Viera et al. reported that only two of seven adolescent ECs (median age 14.6 years, range 12.7–16.6 years) shared the protective HLA Class I type (HLA-B∗27/57/58 : 01/81 : 01) previously described in adult ECs, suggestive of potential distinct, age-relevant immune control mechanisms in pediatric age groups. Studies using nonhuman primate (NHP) models of HIV/SHIV/SIV in adult macaques showed differential impact of antiviral CD8+ T cell depletion between ECs and progressors in chronic and acute infections (22–24). CD8+ T cell depletion resulted in a more prominent increase in viral replication in ECs (22) with a subsequent recovery of viral control upon reconstitution of CD8+ T cells (23), and more profound CD4+ T effector memory cells activation and expansion (24). This finding further highlights the contribution of CD8+ T cells to the suppression of viremia, suggesting that elicitation of effective, polyfunctional antiviral HIV-specific CD8+ T cell responses will be a key for achieving long term viral control in pediatric HIV. NHP model of SIV infection in adult macaques showed viral suppression by CD8+ T cells that recapitulate HIV suppression in ECs (25, 26). These adult macaques were inoculated intravenously with SIVagm.sab92018 and demonstrated viral suppression below the levels of detection of conventional and single copy assays despite persistent reservoirs of replication competent virus. However, it is unclear whether similar characteristics could be achieved in a pediatric NHP model due to differences in early life immune milieus and different route of infection. Therefore, there is a critical need to develop NHP model that can be utilized to understand the mechanisms of viral control in pediatric HIV ECs.

#### CD4+ T Cells

HIV primarily targets CD4+ T cells expressing CXCR4 and CCR5 co-receptors, which undergo dramatic depletion during the acute phase of infection. Even after ART initiation, CD4+ T cell populations do not fully recover (27, 28). Unsurprisingly, PECs were observed to have higher proportion of naïve CD4+ T cells compared to PPs who had an increased effector memory CD4+ T cell population (19). Additionally, immune activation and exhaustion makers on central memory and transitional memory CD4+ T cells were lower in PECs compared to PPs (19). Functionally, HIV-specific CD4+ T cells in PECs are superior to those in PPs, with increased polyfunctionality despite lower activation (19, 29). This phenotype of lower immune activation and exhaustion markers is also strongly established in adult ECs (29, 30). In adult ECs, low HIV DNA viral reservoir is associated with lower activation of both CD4+ and CD8+ T cells (31, 32). However, while the lower activation phenotype is shared between adult and pediatric ECs, they are likely influenced by factors unique to their distinct immune milieus. The lower HIV reservoir and lower activation in CD4+ T cells in adults is attributed to the aggressive CD8+ T cell response in the acute phase of infection (33), while this phenotype in pediatric ECs is preceded by the tolerogenic environment (18). Taken together, these findings demonstrate that the CD4+ T cells in pediatric and adult ECs share similar phenotypes, in which they are less susceptible to infection and exhaustion. However, understanding of mechanisms and pathways that contribute to these phenotypes in the context of tolerogenic early life immunity could potentially benefit immunotherapeutic designs against pediatric HIV.

#### Regulatory T Cells

Tregs are crucial in dampening inflammatory responses, and hence, it is reasonable to hypothesize that Tregs, could be important in slowing progression of HIV pathogenesis. However, there could also be a conflicting dual role of these cells in HIV control. Adult ECs have normal Treg frequencies, with low HIV-specific immune activation while maintaining strong polyfunctional T cell responses (29). A study on pediatric slow progressors (PSP), who are characterized by high CD4+ T-cell counts and low immune activation despite having high viral load, indicated that these PSPs had a higher absolute number of Tregs expressing a suppressive phenotype compared to PPs (34). Additionally, PSPs had higher secretion of the immunosuppressive cytokine IL-10 and a higher frequency of central memory Tregs compared to PPs. Moreover, PSPs that progressed later had lower frequency of suppressive Tregs, lower Treg proliferation, and IL-10 production (34). Together, these findings suggest an active role of suppressive Tregs and anti-inflammatory responses in controlling immune activation thereby slowing progression to disease in children with HIV. Given that the function of Tregs may plausibly limit or promote HIV pathogenesis, more pediatric studies are required to clarify their roles, distributions, and functions in the context of HIV control. Some therapeutic strategies to target Tregs have in fact been studied in adults with HIV. As CTLA-4 on Tregs plays a role in suppressing HIV-specific T cells, blocking CTLA-4 has been proposed as a strategy to rescue an effective HIV-specific T cell response (35, 36). In a study of SIV-infected adult rhesus macaques CTLA-4 blockade resulted in reduced viral RNA in tissues (37). However, another study showed conflicting results when blockade was performed in the early stage of infection (38). Dual monoclonal antibody treatment using anti-PD-1 and anti-CTLA-4 in SIV-infected adult NHPs with long term ART enhanced both memory CD4+ and CD8+ T cells proliferation and effectors functions but was insufficient to control or delay viral rebound after ART interruption (39). Thus, dual CTLA-4/PD-1 blockade is likely insufficient to induce HIV remission and would likely require combination with enhanced killing strategies, such as therapeutic vaccination (40) or coadministration of neutralizing antibodies (41). The potential contribution of Tregs to HIV cure was further demonstrated in NHP model of SIV infection in adult macaques (42, 43). Tregs depletion in SIV infected adult macaques resulted in increased in reactivation of latent reservoirs as well as significant boost of SIV-specific cytotoxic T lymphocytes (CTLs) responses (42). However, Tregs depletion alone is insufficient as a strategy for HIV cure and would require combination with other viral reactivation therapies or vaccination strategies (44). Therefore, future research should focus on refining promising Treg treatments as a strategy for cure in pediatric HIV NHP model.

#### Th17

Th17 cell populations are crucial in maintaining the integrity of the gut mucosa by promoting epithelial cell regeneration as well as coordinating immune responses in this region (45). The absence of these cells results in microbial translocation due to reduced gut integrity, leading to the immune activation which promotes HIV-driven CD4+ T cell depletion, especially in the gut. Consistent with this pathogenic mechanism, studies have shown a higher frequency of Th17 cells in circulation among adult ECs compared to long-term non progressors and even HIV-negative individuals, suggesting a protective role for this cell subset (46). Unfortunately, studies on the distribution and expansion of Th17 cells in HIV infection in children are limited, let alone in the rare group of elite controllers. Dzanibe et al. recently reported that maternal HIV exposure alters the proportions of Th17 and Tregs CD4+ T cells in the periphery of HIV-exposed uninfected infants (HEUs) compared to HIV-unexposed uninfected (HUUs) control infants (47). Additionally, while peripheral Tregs showed inverse correlations with known damage-associated markers in the gut (CCL17, IL-7, CCL20) in HEUs, Th17 cell population was positively correlated with these markers. Taken together, these data suggest that there is an association between markers of gut damage and *in utero* HIV/ART exposure on the HEU infants Th17/Treg balance at birth. However, whether these findings extend to children who acquired HIV from their mothers is unclear.

#### T Follicular Helper Cells

Tfh cells are found in secondary lymphoid organs such as tonsils, spleen, and lymph nodes where ART penetration is not optimal, rendering them sites for ongoing HIV replication (48). Tfh cells have been shown to support HIV persistence, and more interestingly have been demonstrated to be the major HIV reservoirs within central memory CD4+ T cells in adults with chronic HIV infection and on ART (49–51). In adult ECs, however, Garcia et al. demonstrated that peripheral Tfh (pTfh) cells have smaller HIV reservoirs compared to individuals treated with combination ART (52). They postulate a possible link between the low infection of pTfh cells in ECs and their ability to suppress HIV (52). Functionally, pTfh cells in EC’s have been shown to have better B-cell helper activity than those in HIV progressors (53, 54). In children with HIV, the proportion of circulating Tfh cells are shown to be reduced and correlated with memory B cells as well as advanced disease (55, 56). Further research is required to fill the gap in knowledge of the role of Tfh cells in pediatric ECs as this could provide insight on the role of these cells in HIV control and in providing help for improved B cell functions for vaccine design.

### Humoral Immunity

HIV disease progression is associated with phenotypic and functional abnormalities of B cells, several of which are driven by the chronic immune activation associated with HIV replication. However, few studies have evaluated the role of the humoral immune response in spontaneous control of viral replication. In adults, the median frequency of HIV-specific memory B cells in ECs was significantly higher compared to that found in individuals receiving ART (57). While this has not been evaluated in pediatric ECs, a recent study has highlighted B cell dysfunction as a hallmark of HIV disease progression in children with HIV. In this study, a cohort of pediatric long-term non-progressors (LTNPs; n=20) had lower naïve and resting memory B cell frequencies compared to uninfected children (58), while tissue-like memory B cells were lower in LTNPs compared to pediatric progressors. Interestingly, plasma levels of B lymphocyte stimulator (BLyS), a known regulator of B cell function and survival, were lower in pediatric LTNPs compared to progressors. In adults, BLyS expression levels are unaltered, and less B cell dysregulation was observed in non-progressors (59).

Recent work has highlighted a potential role for non-neutralizing HIV-specific antibodies in protection from disease progression and viral control, implicating features that could be exploited in attempts to achieve HIV cure. Muenchhoff et al. described that a subset of ART-naïve children with HIV who maintained normal CD4+ T cells also known as pediatric non progressors (PNPs) had higher p24-specific IgG levels than children under ART, and these anti-p24 IgG were mostly of the IgG1 subclass (60). Meanwhile in PPs, there was a greater contribution to IgG3 subclass to the p24-specific IgG responses. IgG1 mediates antibody responses to viral infection while IgG3 regulates proinflammatory effector functions (61). This suggests that in PNPs viral replication is under control without chronic inflammation, while PPs have more inflammatory responses. Interestingly, when compared to uninfected children, PNPs had no changes in bulk IgG galactosylation, which corresponds to low immune activation and inflammation (60). In PPs, agalactosylated IgG glycans were expanded, while digalactosylated glycans were decreased compared to HIV-uninfected children. Moreover, PNPs demonstrated increased HIV-specific Fc-mediated IgG effector functions, antibody-dependent cellular cytotoxicity (ADCC) and phagocytosis (ADCP), compared to progressors (60). Interestingly, robust ADCC (62, 63) and overall superior polyfunctional Fc dependent activity (64, 65) have been consistently observed in adult ECs. Broadly neutralizing antibodies (bNAbs) arise early with higher breadth and potency in children than in adults with HIV (66–68). While bNAbs in children mostly target similar epitopes to adult bNAbs (69), they also showed remarkable polyclonalities with some bNAbs targeting up to 4 distinct epitopes (69, 70). Altogether, these data suggest that successful monoclonal antibody-based therapeutics for viral remission in pediatric HIV will likely need to exploit neutralizing and non-neutralizing properties of HIV-specific bNAbs to control and mediate clearance of infected cells.

### Innate Immunity

#### Natural Killer Cells

Natural killer (NK) cells are innate lymphocytes that directly kill cells infected by intracellular pathogens, usually viruses. The functionality of NK cells is governed by the interaction of both inhibitory and activating receptors with the MHC-I (71). NK cells are inhibited from killing uninfected cells that have normal levels of MHC-I. The absence of MHC-I molecules causes the loss of inhibitory signals on NK cells, permitting them to directly kill infected cells (71). Genovese et al. demonstrated an association between delayed progression to AIDS and the interaction of protective HLA-B alleles on infected cells and killer immunoglobulin receptors (KIR) on NK cells (72). The interactions between NK cell receptors (KIR3DL1 and KIR3DS1) and the protective HLA-B Bw4-80Ile on target cells are associated with delayed progression to AIDS has been documented (73–75). Interestingly, Tomescu et al. have demonstrated that adult ECs expressing KIR3DL1 on NK cells and HLA-B Bw4-80Ile on target cells had stronger NK cell mediated cytotoxicity compared to CD8+ T cell cytotoxicity from the same individuals (76). These findings suggest an association of NK cell phenotype with development of elite control.

There is a paucity of research on the distribution and function of NK cells in pediatric ECs. Given the immature adaptive immune system in children, NK cell response to infection is an important initial defense. Interestingly, NK cell populations in pediatric long-term non progressors (LTNP) are lower than healthy uninfected individuals but similar to ART-treated children (77). Research on the NK cell population in pediatric ECs is required to identify the distributional and functional correlates of NK cells to HIV control in order to better inform strategies for HIV cure.

#### Dendritic Cells

Dendritic cells (DCs) are innate immune cells are that are specialized in presenting foreign antigens to the adaptive immune system to mount an effective immune response and also release cytokines and chemokines to orchestrate adaptive immune responses. These cells are therefore important in HIV control as they present HIV antigen to T cells to mount effective immune responses. A study showed exposure of conventional DCs to HIV-1 resulted in rapid and sustained production of type I interferon and importantly stronger capacity to stimulate and expand HIV-1-specific CD8+ T cell responses in ECs (78). Furthermore, it was shown that plasmacytoid DCs (pDCs) from ECs had a higher capacity to reduce HIV production compared to those from viremic individuals (79). In adult rhesus chronic SIV infection model, DCs and pDCs influenced ongoing inflammation and T cell exhaustion in the mucosal tissues and there by contributed to persistence of viral reservoirs during ART (80). There is a lack of studies on the role of and functionality of dendritic cell subpopulations in pediatric ECs. During HIV infection, dendritic cells in children undergo numerical and functional deficits. Interestingly, with the administration of ART, the recovery of myeloid dendritic cells was observed whereas that of plasmacytoid dendritic cells was only partial (81).

#### Other Innate Immune Cells

Monocytes are an innate effector cell population which seem to be associated with suppression of HIV viremia. Adult ECs, have overall lower levels of monocyte activation (based on HLA-DR expression) with a reduced frequency of inflammatory monocyte (CD14++CD16+) subset compared to viremic subjects (30). In addition, elite controllers tend to exhibit significantly higher proportion of CD14^+^CD16^+^ monocytes, compared with HIV-negative controls (82).

Innate lymphoid cells (ILCs) are lymphoid-lineage cells that are further classified as ILC1, ILC2, and ILC3 based on the transcription factor they express, and their functions overlap with that of CD4+ T helper (Th)1, Th2, and Th17 lymphocytes, respectively. ILCs are severely depleted in the circulation of HIV-infected children however, initiation of ART at birth preserves ILC development and function (83). While a role for ILCs has not been established in elite controllers, the aforementioned study establishes a connection between the maintenance of ILCs in population and control of HIV replication in pediatric populations.

Gamma delta T cells (γδT) are a subset of innate-like T-cells with a transcriptional phenotype that blends characteristics of both NK and CD8+ T cells, thus exhibit highly cytotoxic activity (84, 85). Gamma delta T cells represent a small fraction (1-5%) of the overall T cell population but are more abundant in barrier sites like the skin, lung, digestive tract, and reproductive organ mucosa (86). Recent work demonstrated that interleukin (IL)-17-production by γδ T cells was better preserved in PBMCs from adult ECs than in untreated HIV-infected patients and was negatively associated with immune activation (87). Moreover, overall alterations in γδ T-cells were less prominent in ECs compared to untreated HIV- infected adults. The extent to which γδT cells contribute to natural control of HIV infection in children and adults remains elusive, however a few studies have highlighted the ability of γδT cells to inhibit HIV replication *in vitro* (88, 89).

### Summary

There are several opportunities in the early life immune system that could be exploited towards an HIV cure. The immunotolerant and naive immune environment can allow low levels of activation and immune system maturation is highly adaptable. A better understanding of the early life immune ontogeny and mechanism of viral control in pediatric ECs could provide insight into novel immune-based strategies towards clearance of latently infected cells and viral remission in children with HIV.

## HIV Reservoir in Pediatrics

### Composition of the Latent Reservoir in Adults and Children

The latent HIV reservoirs are heterogenous in both cell types and tissue localizations within the body. Various CD4+ T cell types are susceptible to HIV infection including naïve cells, long-lived memory cells (stem cell and central memory cells), and terminally differentiated cells (90), but central memory cells CD4+ T cells are the most vital to the maintenance of the latent reservoir (91). HIV-infected CD4+ T cells exist broadly throughout the body and have been found in the gut-associated lymphoid tissue, genital tract, and lymph nodes (92). In addition to CD4+ T cells, myeloid immune cells have been implicated as HIV reservoirs. Macrophages are known to be infected by HIV (91, 93) and *in vitro* infectivity studies showed that they are not killed by HIV, suggesting that they are likely HIV reservoirs (93). Tissue resident macrophages are highly specialized cells and varied in susceptibility to HIV infection. Alveolar macrophages and microglial cells are susceptible to HIV infection and contribute to the formation of cell reservoirs in the lung and brain, respectively (94, 95).

The majority of studies that focused on kinetics and origin of persistent latent reservoirs that eventually contribute to viral rebound when ART is interrupted were conducted in adults with HIV or adult NHPs. Infant NHPs are highly relevant and translatable models for the study of pediatric HIV infection and persistent latent reservoirs. Several studies using oral infant NHP Simian-Human Immunodeficiency Virus (SHIV) infection model that mimic breastmilk transmission of HIV, the gastrointestinal (GI) tract was identified as major source of viral RNA during ART and upon viral rebound (96–98). Following ART discontinuation, while the GI tract showed the most rapid increase in viral RNA, virus was also detected in the nasal-associated lymphoid tissue (NALT), axillary lymph node, and spleen (97). An earlier study described that while the oral mucosa and tonsils are generally the anatomical location for initial establishment of a small founder population of infected cells, the anatomical location of persistent latent reservoirs in SHIV-infected infant NHPs continues to spread into distal lymph nodes and other organs including the brain, lungs, intestines, pancreas, and spleen (99).

In addition, due to differences in age-related immune landscapes, the latent HIV reservoirs in adults and children are defined by distinct characteristics. In perinatally-acquired HIV in children, the latent reservoir consists of transitional memory CD4+ T cells as opposed to the mostly central memory CD4+ T cells observed in adults (100, 101). Additionally, perinatally-infected children have the unique opportunity of initiating ART early, which has been reported to limit the establishment of the initial latent HIV reservoir (12, 102, 103). A recent study demonstrated that reactivation of latent HIV reservoirs with PMA/ionomycin from adolescent who were perinatally-infected and treated within the first 24-months of life, resulted in similar rate of cell activation but slower and lower magnitude of proviral load when compared to infected adults (104), suggesting that latent viral reservoirs in children were more resistant to *ex vivo* reactivation. This study highlights differences in inducibility of latent viral reservoirs resulting from perinatal versus adult HIV infections, which could impact strategies toward latent reservoirs clearance and viral remission in children.

### Immune Signatures of the Viral Reservoir

CD4+ T cell immune signatures associated with the size of viral reservoir in adolescents and children who were vertically infected with HIV were recently described (105). Rinaldi et al., reported that while co-expression of PD-1 and TIGIT was associated with CD4+ T cell viral reservoir in both children and adult, the frequencies of PD-1 and TIGIT expressing CD4+ T cells along with HIV-specific CD4 T cells were able to discriminate between lower and higher viral reservoirs in perinatally-infected children (105). This finding suggests that while some immune signatures that could be targeted by interventions or therapies towards clearance of latent viral reservoirs are similar in children and adults, there are also unique signatures that immune-based strategies aimed at permanent HIV remission in children could target.

## Current Immune-Based Therapies Potential for Pediatric HIV Viral Remission and Cure

Elimination of latently infected cells that are established early during infection remains as a major challenge to cure in children and adults living with HIV (8, 106, 107). While ART treatment can achieve sustained viral suppression, it does not eliminate latently infected cells, which lack active expression of viral proteins on their cell surface and therefore are invisible to the immune system. ART discontinuation reactivates latently infected cells harboring intact replication competent HIV provirus to produce HIV virions, resulting in sustained viral rebound in nearly all individuals with HIV (106–108). This indicates that the host immune system of most individuals with HIV is unable to eliminate virus producing and/or infected cells in persistent reservoirs. Therefore, novel strategies with combinatory effect to induce viral production by latently infected cells while on ART and to augment the ability of host immune responses in clearing infected cells are paramount to HIV cure. Additionally, as the majority of these novel interventions are currently developed in adults with HIV or preclinical models for adults, it is imperative to design novel combination strategies that are tailored to the developing immune system in children for eradication of pediatric HIV.

### Therapeutic HIV Vaccines (T and B Cells)

The goal of therapeutic vaccination is to improve the functional capacity of the host immune system to kill infected CD4+ T cells and/or neutralize circulating viruses (109). Several therapeutic HIV vaccines have been assessed preclinically and clinically for their safety, immunogenicity, and effectiveness but these studies have largely focused on adult HIV population or SIV/adult nonhuman primate (NHP) models (107, 109). It is well established that perinatally-infected infants and children who are treated early with ART tend to have a small pool of latent viral reservoir, normal development of B and T cell compartments, and a high capacity to regenerate an immune repertoire (110, 111). While lessons from the “Mississippi baby” have indicated that early ART treatment is not enough to achieve life-long HIV remission, such characteristics do render perinatally-infected children as an ideal group to test therapeutic vaccines for functional HIV cure (110, 112). However, to date, only two clinical and one preclinical NHP therapeutic HIV/SIV vaccine studies have been conducted with a focus of HIV cure in infants and children (**Table 1**).

**Table 1:** Therapeutic HIV/SIV vaccine studies for pediatric HIV cure.

Key parameters	Clinical		Pre-clinical
Study/Trial	ACTG218 (NCT00000762)	PEDVAC (NCT04301154)	–
Infectious agent	HIV-1	HIV-1	SIVmac251
Therapeutic vaccine compound	Env viral proteins; gp160 and gp120	HIVIS DNA (HIV-1 subtype A, B and C, encoding Env, Gag, Rev, RT)	Ad48-SIV, MVA-SIV and GS-986
Study population	Infants and children	Children	Infant RMs
Age	1 month to 18 years	6 to 16 years	1 month
Vaccination schedule	weeks 0, 1, 2, 3, 4, 6	weeks 0, 4, 12 and 36	week 22, 30, 38 and 50
Immunogenicity	Moderate to strong antigen specific antibody response. Vaccine antigen specific lymphoproliferative responses	Compared to aged match controls, higher HIV gag-specific cellular immune response. Lymphoproliferative response to the gag virion antigen (HIV-1 MN) was higher in children than in adults	Greater magnitude and breadth of polyfunctional CD4 and CD8 T cells in vaccinated RMs. Levels of SIV specific gp120 specific antibodies were significantly boosted following MVA vaccination. No effect on viral reservoir or rebound

The Pediatric AIDS Clinical Trials Group 218 (ACTG 218 or NCT00000762) was the first clinical study conducted to evaluate the safety and immunogenicity of viral envelope proteins as therapeutic vaccine in infants and children with HIV. The rationale behind this study was that immunization with HIV envelope (Env) protein-based vaccines could alter the landscape of naïve immune responses to HIV and thereby delaying viral rebound. Three HIV Env-based vaccines were administered to infants and children with HIV aged 1 month to 18 years at the time of entry and then at 1, 2, 3, 4 and 6 months later. The vaccines were well tolerated with no adverse effects, secondary infections, or change in viral replication status. Around 65% of vaccinees demonstrated moderate to strong antigen-specific antibody responses and up to 56% of vaccinees exhibited vaccine antigen-specific lymphoproliferative responses, indicative of immunogenicity of the therapeutic vaccine (113).

The First Pediatric Randomized Trial (PEDVAC or NCT04301154) evaluated safety and immunogenicity of the multiclade, multigene HIV DNA therapeutic vaccine (HIVIS) in 10 HIV infected and ART treated children of 6 to 16 years of age. Administration of an HIV DNA vaccine construct expressing HIV-1 subtypes A, B, and C, Env, Rev, Gag, and reverse transcriptase (RT) was well tolerated and exhibited a safe profile except for local irritation, erythema with or without swelling or usual itching at the site of injection. Unlike, non-vaccinated children, vaccinees exhibited increased percentage of HIV-specific perforin-producing CD8+ T cells, compared to baseline, highlighting the immunogenicity of therapeutic HIV-DNA vaccines (114–116). However, there was no evidence that the vaccine had an impact on latent viral reservoir and viral rebound.

Preclinical studies demonstrated the promise of therapeutic vaccination with Ad26/MVA (recombinant adenovirus serotype 26 (Ad26) prime, modified vaccinia Ankara (MVA) boost) encoding SIV gag, pol, env in combination with a toll-like receptor 7 (TLR-7) agonist as an adjuvant against Simian Immunodeficiency Virus (SIV) using an adult NHP model (117). This vaccine regimen was recently translated to an infant SIV NHP model that simulated postnatal HIV infection through breastfeeding with ART-mediated suppression of viremia (118). In this study, a total of 8 infant NHPs were orally infected with SIVmac251 and treated with ART at 4 weeks post-infection followed by multiple immunization with Ad48/MVA encoding SIV gag, pol env plus TLR7 while remining on ART. The vaccinated group showed greater magnitude and breadth of polyfunctional CD4+ and CD8+ T cells when compared to control group and, as anticipated, the administration of the TLR-7 agonist (GS-986) led to activation of monocytes and T cells. This finding was similar to the observations found in adult NHPs (117). Additionally, the levels of SIV gp120-specific antibodies were significantly increased following the MVA boost. However, despite these robust vaccine-induced cellular and humoral immune responses, the size of viral reservoirs, time to viral rebound, and viral set point following analytical ART interruption (ATI) were similar in the vaccine and control groups. These findings suggest that while therapeutic vaccine-induced immunity may induce antiviral T and B cell responses, these responses may be insufficient to modulate viral rebound dynamics during ATI. Thus, there is a need to identify T and B cell responses that could potentially impact the latently infected HIV reservoir and to combine therapeutic vaccines with other immune therapies such as potent latency reversing agents (LRAs), passive transfer of broadly neutralizing antibodies (bNAbs), and check point blockade if this approach is pursued as strategy for HIV remission in children.

## Passive Immunization Strategies

### Broadly Neutralizing Antibodies

Recent development in the use of antibodies for HIV therapy showed promise in the ability of antibody-based strategies to reduce plasma viremia and to mediate clearance of infected cells to further delay viral rebound following ATI (119–123). Emerging evidence supports the effect of antibody-based therapies on persistent latent HIV reservoirs, which could potentially induce viral remission when used alone or in combination with ART and other novel therapeutic modalities (121).

Passive infusion with highly potent broadly neutralizing antibodies (bNAbs) against distinct HIV Env regions may represent an advantageous strategy due to the ability of bNAbs to neutralize free virions *via* the Fab domain and to engage cognate receptors on host innate and adaptive *via* the Fc domain. Passive infusion with single or combination bNAbs is actively evaluated as therapeutic strategy for HIV remission in the clinics by measuring the effects of antiviral activities of bNAbs on plasma viremia and viral suppression following ATI as well as impact on viral reservoir by delaying viral rebound (121). Most of these studies were conducted in adults with acute or chronic infections while on ART and similar studies in pediatric HIV population are lacking. To date, only two ongoing clinical trials of bNAb therapy as part of an HIV treatment strategy have been conducted in infants and children (**Table 2**). Passive infusion of the unmodified version and the long acting (LS) version of the CD4 binding site (CD4bs), VRC01, was shown to be safe and well tolerated in HIV-exposed infants (124, 125). Similarly, passive infusion of the modified and extended half-life version of the VRC01 with the LS mutation in combination with the V3-glycan bNAb, 10-1074, was also safe and well tolerated in the pediatric population (125). The ability of monotherapy and dual therapy using bNAbs to maintain viral suppression and delay viral rebound following ATI in the pediatric HIV population is still under investigation.

**Table 2:** Antibody (bNAb) therapy for pediatric HIV treatment.

Key parameters	Clinical	
Study/Trial	NCT03208231 (IMPAACT 2008)	NCT03707977 (Tatelo Study)
BNAb/combination	VRC01	VRC01-LS, 10-1074
HIV Env target site	CD4 binding site	CD4 binding site, V3 glycan
Primary purpose	Treatment	Treatment
Dosing	Week 0, 2, 6, and 10	Week 0, 4, and 8
Study population	HIV-infected infants on ART	Antepartum or peripartum HIV-infected children with early ART initiation
Study endpoint	Safety, pharmacokinetics, effects on plasma viremia	Safety, pharmacokinetics, impact on viral rebound

Preclinical data from SHIV infection in adult NHP models demonstrated that administration of single or combination bNAb therapy could potentially target, control, and eliminate viral reservoir, suggesting that ART free remission is attainable following ATI (126–132). These mono- or dual-bNAb therapies were administered either alone or in combination with other interventions strategies, including therapeutic vaccines (126, 127) and immune modulatory agents (130–133). Depletion of CD8+ T cells resulted in transient increase of plasma viremia in animals with controlled viral suppression, suggesting that mechanism of viral control is likely co-dependent on CD8+ T cell immune responses (126, 127). The potential immunomodulatory effect of bNAb therapy on CD8+ T cells was recently demonstrated in adults in the clinic, in which administration of combination bNAb therapy (3BNC117 and 10-1074) was associated with an increase in Gag-specific CD8+ T cells in the blood (134). However, whether the increase in HIV-specific CD8+ T cell immune responses contribute to viral control and suppression in the absence of ART remains to be seen. Taken together, these studies highlight the potential of bNAb therapy as an alternative or adjuvant to ART in mediating viral control and/or augmenting the host endogenous immune responses for viral control. Given the unique characteristics of early life immunity as discussed above, it is imperative that effort to advance the use of bNAb therapy in pediatric HIV be tailored to the developing immune system cell composition, activation state, and milieu. Indeed, the highly tolerogenic and anti-inflammatory immune environment during early life as well as differences in pathogenesis between adults and children with HIV may influence efficacy of bNAb therapy towards viral control and remission the pediatric HIV population (135). Thus, the endogenous immune responses in pediatric HIV could potentially be harnessed towards viral elimination and clearance of persistent reservoir to achieve viral remission in the absence of ART. Future studies should aim to investigate the effect of bNAb therapy alone or in combination with therapeutic vaccines and other immunomodulatory agents in augmenting endogenous immune responses as a potential strategy for HIV cure in the in the context of early life immunity.

Numerous efforts are currently being developed to ensure consistent high levels of bNAbs in the circulation including various modifications to extend their half-lives (120, 136–139) and expression of bNAbs using viral vectors (122, 140, 141). Additional strategies are currently being developed to enhance the ability of Fc effector functions, including antibody-mediated cell cytotoxicity (ADCC) and antibody-dependent cell phagocytosis (ADCP), to clear HIV-infected cells (121, 122, 142–144). The Fc region enhancement can be accomplished by (i) altering the antibody isotype (142, 143), (ii) modifying the antibody Fc domain amino acid sequences (139, 144), and (iii) modifying the antibody Fc glycans (122, 145). These approaches modulate antiviral breadth and potency of bNAb therapy by increasing bNAbs affinity to cognate Fc receptors on host immune cells and host complement proteins for elimination of HIV-infected cells. These current novel modalities represent tremendous opportunities for pediatric HIV cure. Passively acquired and endogenous ADCC-mediating antibodies contributed to better disease outcome in infants with HIV (146–149). This suggests that passive immunization with bNAbs exhibiting enhanced antiviral functions could be especially attractive for pediatric HIV treatment.

### Engineered Antibody-Like Molecules

One major challenge in bNAb therapy is the emergence of viral escape to one or more bNAbs due to selection or mutation once the plasma levels of passively administered bNAbs start to decline (150). Antibody engineering through generation of bi- (151, 152) and tri-specific (153–155) bNAbs against HIV have been demonstrated to increase potency and breadth of combination bNAb therapy. An attractive option to improve bNAb performance and overcome their intrinsic limitations is the development of antibody-like molecules, in which antibodies are engineered to (i) improve affinity and recognition of viral epitopes, (ii) combine bi- or tri-specificities, and/or (iii) modify Fc domains to improve half-life and increase engagement of effector cells (156). Overall, there are two major classes of bi- or tri-specific antibody-like molecules: those with or without an antibody Fc region (157).

Asokan and colleagues constructed four different bi-specific immunoglobulins (IgGs) composed of independent antigen-binding fragments with a common Fc region: VRC07×10E8, VRC07×PGT121, VRC07×PG9-16, and 10E8×PG9-16. All four bi-specific IgGs neutralized over 94% of antigenically diverse viruses in a panel of 206 HIV-1 strains, with VRC07xPG9-16 showing the most positive neutralization profile (158). In a step further in the development of these molecules, a tri-specific antibody-like molecule was generated to include bNAbs with 3 HIV Env specificities, N6 (targeting the CD4bs), PGDM1400 (targeting the V2 glycan), and 10E8v4 (targeting the MPER). This tri-specific antibody-like molecule neutralized clade A SHIV BG505 more potently than any of the parental bNAbs (154), showing comparable potency but greater breadth than a previously developed tri-specific molecule including VRC01/PGDM1400/10E8v4 specificities (153). The newly improved tri-specific antibody-like molecule (N6-CD4bs/PGDM1400-V2 glycan/10E8v4-MPER) was shown to reduce viremia from 100- to 1000-fold in viremic SHIV-infected adult NHPs (154) although transient viral rebound was observed when ART was interrupted, likely due to decline of antibody-like molecule level. However, similar to prior observations with mono- and dual-bNAb therapy, rebound viremia was returned to low levels *via* CD8+ T cell mediated viral control (154).

Another interesting approach to engineer antibody-like molecule against HIV involves combining a domain to target the virus protein and another domain to target host cell receptors. Mainly, two different constructs have been developed following this strategy: Bi-specific T/NK cell engagers (BiTEs/BiKEs) and Dual Affinity Re-targeting Molecules (DARTs). BiTEs are composed of antibody domain targeting the HIV-1 Env protein gp120 through B12 or VRC01 and an anti-human CD3 single chain variable fragment (scFv) was shown to redirect lysis of HIV gp120-transfected CHO cells *in vitro*, as well as inhibited HIV replication in both HIV-infected PBMCs and macrophages cocultured with autologous CD8+T cells (159). Similarly, BiKEs are composed of antibody domains D6 and E11, which bind to human CD16 present on NK cells, and a soluble CD4 domain, which binds to HIV gp120. BiKEs activate NK cells in the presence of target cells in and mediate infected target cell lysis (160). DARTs are novel bi-specific-based constructs that seem to offer improved stability, manufacturability, and potency compared to the BiTEs (161). The variable domains of the two antigen-binding moieties in DART molecules are incorporated into a disulfide-linked heterodimer, stabilizing the structure. DART molecules with HIV-1 Env and CD3 specificities were evaluated for their capacity to recruit and redirect cytotoxic T effector cells to primary HIV infected CD4+ T cells.

Unfortunately, all studies with bi/tri-specific molecules so far focused on cells from adult participants, leaving unknown the potential as therapy for pediatric infection. Importantly, immune system development during early life will probably affect the therapeutic potential of bispecific molecules, and thus children’s effector cells might have improved activity profiles.

### Latency Reversing Agents

Latency reversing agents (LRAs) are small-chemical compounds employed to activate and expose latently HIV-infected cells to the host immune system (92). LRAs target different mechanisms of HIV transcription and replication. The current classes of LRAs induce epigenetic modification, sequester transcription factors, and target the HIV Tat protein (162). Among the epigenetic LRAs, there are: histone deacetylase inhibitors, histone methyltransferase inhibitors, and bro- and extra terminal domain inhibitors. These drugs act at the HIV-1 LTR promoter and induce histone modifications at the chromatin levels, which prevent RNA polymerase from initiating transcription. There are also single-agonists LRAs like protein kinase C (PKC), that induce HIV latency reversal in model systems, which has broad impact activity and could potentially have dangerous side effects *in vivo*. The potential broad side effects of LRAs are one of the major challenges in employment of LRAs in the clinic for pediatric HIV treatment.

Many anti-tumor drugs approved for use in adults and children are currently under evaluation for their potential to act as LRAs in adults with HIV (**Table 3**). Existing anti-tumor drugs are easily translatable to LRA discovery because of the shared strategy to induce epigenetic changes that promote cell cycle arrest, apoptosis, autophagy, and cell death of cancer-infected cells (163). Several LRAs have undergone testing in adult clinical trials including the histone deacetylase inhibitors, such as vorinostat, panobinostat, belinostat, and romidepsin, as well as immunomodulatory compound such as IL-15 receptor superagonist complex (N-803). While some drugs have induced HIV-1 transcription and T-cell activation, no candidates have effectively reduced the size of the virus reservoir (162). Clinical trials in adults with HIV and treated with ART in combination with romidepsin and vorinostat showed increased levels of plasma HIV RNA and cell-associated unspliced RNA in CD4+ T cells respectively. However, no significant changes in virus reservoir size were observed (164, 165). Recently, a regimen of vorinostat along with hydroxychloroquine and maraviroc was reported to increase the levels of plasma HIV RNA in a cohort of adults on ART but no change in the virus reservoir size was detected (166). In addition, upon ART interruption, no difference in time to viral rebound between the vorinostat and control groups was observed (166). Miller et al. reported that escalating doses of the investigational drug N-803, an IL-15 receptor superagonist complex (IL-15/IL-15α-Fc) was safe and well-tolerated in adult living with HIV in Phase 1 clinical trial (167). However, future investigation is still needed to determine the impact of N-803 on HIV reservoirs.

**Table 3:** Latency reversing agents in development for adults and for potential use in pediatric HIV treatment.

Key parameters	Clinical					Pre-clinical		Clinical			
Trial information	NCT04340596		NCT02092116	NCT01365065	NCT02475915	–	–	NCT04341311	NCT04897880	NCT00217412	NCT01422499
	Phase I		Phase I/II			–	–	Phase I	Phase II	Phase I	Phase I/II
Infectious Agent/Disease	HIV	HIV	HIV	HIV	HIV	SIV	HIV	Diffuse intrinsic pontine glioma	Solid tumors	Solid tumors	Solid tumors
Drug class	IL-15 Receptor Superagonist	HDACi	HDACi	HDACi	HDACi	SMAC-mimetic	HDACi	HDACi	HDACi	HDACi	HDACi
Drug	N-803	Romidepsin	Romidepsin; with HIV immunizations	Vorinostat	Vorinostat, with hydroxychloroquine and maraviroc	AZD5582	Panobinostat	Marizomib	Panobinostat	Vorinostat	Vorinostat
								Panobinostat			
Dose	0.3, 1.0, 3.0 and 6.0 mcg/kg	5 mg/m2	5 mg/m2; 12 mg/mL Vacc-4x and 0·6 mg/mL rhuGM-CSF	400mg daily	400mg daily	–	2mg/kg	5 mg/m^2	10mg/m2	180 mg/m2	230 mg/m2
Study population	Adulta	Adults	Adults	Adults	Adults, ART interrupted	Adult Rhesus macaque; adult BLT humanized mice	Adult BLT humanized mice	Children	Adult; Children	Children	Children
Age	> 18 years of age	–	> 18 years of age	> 18 years of age	> 18 years of age	–	–	< 22 years of age	< 39 years of age	12 months - 22 years of age	3-18 years of age
Summary	Safe and well tolerated, modest reduction in the inducible reservoir in PBMCs that persisted for up to 6 months	Increased plasma HIV-1 RNA, but did not change reservoir size	Therapeutic HIV immunization followed by romidepsin, resulted in decline in total HIV-1 DNA	Increased cell-associated unspliced RNA in CD4+ T cells, but did not change plasma HIV RNA	Increased plasma HIV RNA, but did not change reservoir size	Latently infected cells activated through the NFkB pathway and induced HIV-transcription	Histone acetylation broadly observed, but no detectable change in the HIV RNA, DNA, or latently infected resting CD4 + T cells	Ongoing	Ongoing	Vorinostat is well-tolerated at 180 mg/m2 in children and comparable to adults	Higher doses than 230mg/m^2/day correlated to tumor response and longer progression free survival

Currently, there are no clinical trials for evaluation of LRAs in pediatric HIV populations. However, several of the LRAs in clinical trials for adults have also been evaluated as anti-tumor drugs for pediatric cancer treatment (**Table 3**). The clinical trials of vorinostat and panobinostat in pediatric cancer populations showed that the drugs were well-tolerated, suggesting the potential opportunity to safely translate these drugs into LRAs to the pediatric HIV population (166, 168). Preclinical studies in adult rhesus and humanized mouse models showed promising results in using the mimetics of the second mitochondrial-derived activator caspases (SMAC) to activate latently HIV-infected cells *via* the non-canonical NF-Kb pathway, in which increased levels of SIV-RNAs were observed in resting CD4+ T cells derived from tissues following SMAC mimetic (AZD5582) treatment (169). However, a more recent study by Bricker et al, reported that while the pharmacokinetics and pharmacodynamics (PKPD) study of repeated AZD5582 dosing was safe in preclinical infant rhesus macaques, the investigational drug showed weaker latency reversing activity in infant than in adult rhesus macaques, likely due to altered pharmacokinetics and less inducibility of infant CD4+ T cells (170). Therefore, additional studies in both clinical and preclinical models to investigate the impact of novel latency reversing strategies, in the context of developing immune system, on latent viral reservoir reactivation and their combination with anti-viral immunotherapeutic strategies would highly benefit the quest for pediatric HIV cure.

### Summary

The eradication of persistent latently infected HIV reservoirs will likely require combinations of multiple immune-based interventions, whether alone or in combination with ART. In addition to the treatment regimen composition, dosing and timing will likely be key elements in efforts to limit initial establishment of a founder population of infected cells or to purge latently infected cells harboring replication competent HIV provirus in children with HIV.

## Conclusions

The unique context of early life immunity and the distinct nature of persistent viral reservoirs in pediatric settings suggest that pediatric-specific strategies will likely be required to achieve ART-free remission in children living with HIV. ART alone is insufficient and a combination of immune-based interventions that can directly target and/or augment host immune effector functions will probably be needed to eliminate latently HIV-infected cells. However, many of the recent advances in understanding HIV persistence and strategies to eliminate latent viral reservoirs for HIV cure using immune-based strategies are conducted in adults, and there is a paucity in studies involving children living with HIV. Current HIV cure strategies cannot be directly extrapolated to children due to (1) unique differences in early life and adult immune systems, (2) the distinct kinetics of latent virus reactivation in adult and children, and (3) differences in route of infection or transmission of HIV in adult and children. Immune-based intervention strategies such as therapeutic vaccines that target T and/or B cells or passive immunization using bNAbs or engineered Ab-like molecules in combination with LRAs will likely need to be adapted to the pediatric setting. Additionally, these intervention strategies will likely need to be tailored to both perinatal and postnatal HIV infections in children. ART interruption poses the risk of viral rebound, emergence of resistance strains, and other unknown risks (171). However, a study in small subset of perinatally infected infants (7 infants, age <12 weeks) treated early with ART, planned treatment interruption resulted in prolonged viral control (median of 57 weeks off treatment) and that restarting of treatment led to improvement in CD4+ T cell counts (171).

One of the major limitations of HIV cure research in the pediatric population is the ethical and risk-to-benefit considerations around the optimum time to interrupt treatment in order to assess successful eradication or prevention of long-term HIV reservoirs establishment (172, 173). The unique immune profiles of pediatric ECs could provide better understanding of the immune mechanisms involved in long term viral control in the absence of treatment and may guide the development of immune-based strategies targeted towards augmentation of infant early life immune responses for eradication of latent HIV reservoirs.

## Future Directions

The use of infant NHP models for both perinatal and postnatal pediatric HIV infections in the context of ART is crucial in advancing our understanding of the persistent latent HIV reservoir and how it can be targeted in the unique setting of early life immune development. Additionally, development of infant NHP models that recapitulate the slower disease progression in pediatric ECs in the absence of treatment would be highly valuable in investigating the immune mechanisms involved in viral control and development of immune-based strategies for pediatric HIV cure.

## Author Contributions

SB, AN, BY, RG, TS, and CG conceived, designed, and wrote the article. CP, SD, MN, and AB contributed to writing part of the article. GF, AC, SP, and RA contributed to the conception, design, review and editing of the manuscript.

## Funding

This work was supported by grant P01 AI131276 from the (National Institute of Allergy and Infectious Diseases (NIAID) to AC, GF, and SP. AC, GF, SP are also supported by UM1 AI164566. SB is supported by the Interdisciplinary Research and Training Program in AIDS (5T32AI007392-31), Duke University.

## Conflict of Interest

Author SP serves as a consultant for Moderna, Merck, Dynavax, Pfizer, and Hoopika CMV vaccine programs and leads a sponsored research program on CMV vaccines with Moderna and Merck.

The remaining authors declare that the research was conducted in the absence of any commercial or financial relationships that could be construed as a potential conflict of interest.

## Publisher’s Note

All claims expressed in this article are solely those of the authors and do not necessarily represent those of their affiliated organizations, or those of the publisher, the editors and the reviewers. Any product that may be evaluated in this article, or claim that may be made by its manufacturer, is not guaranteed or endorsed by the publisher.
